# Analyzing the Impact of COVID-19 on the Well-Being of Pharmacists: A Narrative Review

**DOI:** 10.7759/cureus.66804

**Published:** 2024-08-13

**Authors:** Sainamitha R Palnati, Saajan H Bhakta, Pooja Patel, Bradley J Newell

**Affiliations:** 1 Department of Research, Kansas College of Osteopathic Medicine, Wichita, USA; 2 Department of Community Pharmacy, Dandurand Drugs, Wichita, USA; 3 Department of Pharmacy Practice, University of Kansas School of Pharmacy, Wichita, USA

**Keywords:** psychological well-being, psychological stressors, pharmacy, pharmacists, midwestern usa, mental health, kansas, covid-19 pandemic, burnout

## Abstract

The COVID-19 pandemic impacted the mental, emotional, and physical health of healthcare professionals around the world. Despite research efforts to examine the experiences of physicians, nurses, and other professionals in healthcare, little has been shared about the experiences of pharmacists during the pandemic. A review of current evidence is needed to better understand the mental health experiences of pharmacists during the pandemic.

This review aimed to understand the perceptions, experiences, and impacts on the mental, emotional, and psychological well-being of practicing pharmacists during the COVID-19 pandemic.

A review of the literature was carried out by searching several electronic databases, which included Google Scholar, ScienceDirect, PubMed, DOAJ, JSTOR, PsycINFO, the ERIC Database (via EBSCOhost), and Academic Search Complete for studies published between 2020 and 2022. The search was conducted from September 10, 2022, to November 18, 2022, using Boolean operations and the following terms: Kansas, pharmacist, pharmacy, mental health, psychology, burnout, and well-being.

Nine studies exploring the lived experiences of practicing pharmacists worldwide during the COVID-19 pandemic were included in this review. The literature surveyed includes three international studies, five national (USA) studies, one study from the Midwestern region of the USA, and one study from Kansas specifically. These studies revealed increased burnout prevalence, mental health distress, feelings of negativity related to the job, and an overall lack of wellness among pharmacists across the world during the pandemic.

There is limited research on the perspectives and experiences of practicing pharmacists during the COVID-19 pandemic. Additional research is necessary to thoroughly understand pharmacists' experiences and how to further support their well-being.

## Introduction and background

COVID-19 is caused by the SARS-CoV-2 virus, with the first case identified in Wuhan, China, in 2019 [1]. Since the World Health Organization declared this a global pandemic, its impact has compromised day-to-day life. The repercussions are profound, particularly in the USA, where healthcare workers, including pharmacists, face heightened risks of burnout and mental health challenges [2-6].

The strain on healthcare delivery, coupled with frequent workplace changes such as staffing shortages, new technology implementations, and policy updates, has led to a surge in burnout among pharmacists globally, despite the compassion satisfaction achieved through caring for patients at that time [6-8]. The increased workloads created stressors related to family, financial sustainability and housing, and personal health [8].

In an effort to help the healthcare community learn and support colleagues, this review of the mental health impact on pharmacists during the COVID-19 pandemic is organized geographically, starting with international studies briefly analyzed to identify potential methodologies for future research. Next, it narrows the scope to the national level, highlighting the widespread impact of the pandemic on practicing pharmacists in the USA. Additionally, this review directs attention to the Midwestern region of the USA, with a specific focus on Kansas, to emphasize the unique challenges faced by pharmacists in rural settings within the state.

This study was presented as a poster presentation at the 2023 Kansas Association of Osteopathic Medicine Mid-Year CME Conference on November 10th, 2023, and at the 2023 Kansas College of Osteopathic Medicine Research Day on May 5th, 2023. Additionally, the work was presented in an invited symposium at the 2023 American Psychological Association Annual Convention in Washington, D.C. on August 2nd, 2023. This work was presented as a poster presentation at the Pharmacy Education 2024: American Association of Colleges of Pharmacy annual meeting on July 21st-22nd, 2024, in Boston, MA.

## Review

Methods

**Figure 1:**
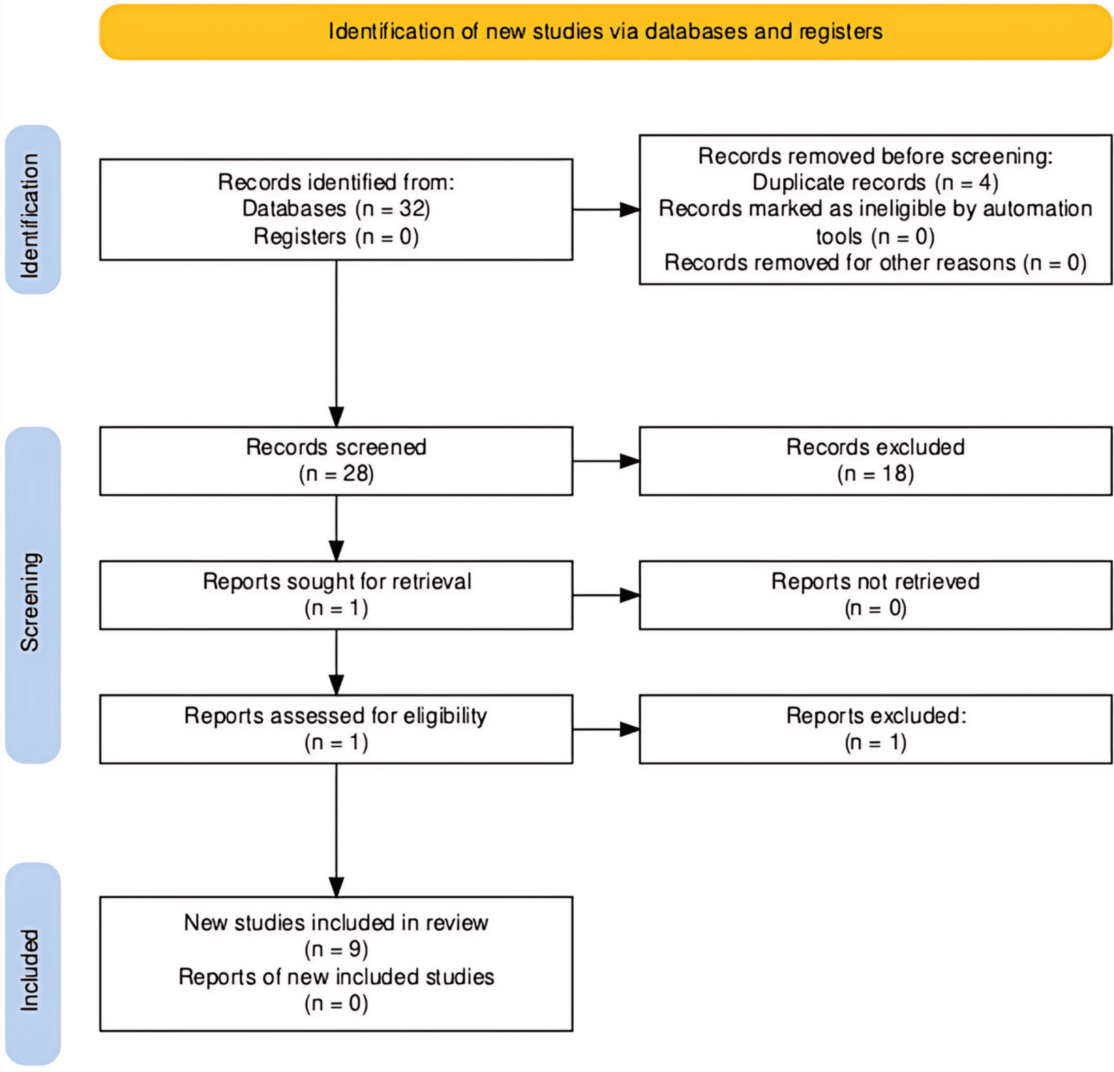
PRISMA flow diagram of the summarized search strategy n: number of studies, PRISMA: Preferred Reporting Items for Systematic Reviews and Meta-Analyses

To compile this review, electronic databases were searched from September 1, 2022, to November 28, 2022, which included Google Scholar, ScienceDirect, PubMed, DOAJ, JSTOR, PsycINFO, ERIC Database (via EBSCOhost), and Academic Search Complete. The search utilized keywords including Kansas, pharmacist, pharmacy, mental health, psychology, burnout, and well-being. Boolean operators were employed for a comprehensive search. The inclusion criteria for this review were peer-reviewed studies published in the last four years, research involving pharmacists, and specifically focused on mental health and well-being during and after the COVID-19 pandemic. The exclusion criteria consisted of studies conducted before the COVID-19 pandemic, case reports, non-peer-reviewed studies, and non-English publications. To minimize bias, all identified records were screened and agreed upon by the entire research team of one psychologist, two practicing pharmacists, and one medical student. Nine relevant publications within the 2020-2022 timeframe were included and synthesized into the following sections: (i) international, (ii) national: USA, (iii) regional: Midwestern USA, and (iv) Kansas. Figure 1 presents a flow diagram of the search strategy utilized for this review.

Literature review

To understand the experiences of pharmacists during the pandemic, a thorough review of literature on the international, national, and regional (Midwestern USA) levels was necessary. The literature underscored how significantly the pandemic impacted the mental, emotional, and psychological health of pharmacists.

International

In Australia, Johnston et al. analyzed the prevalence of burnout and described the work and psychosocial factors affecting male and female pharmacists during the pandemic. The methodology consisted of an online survey using the Maslach Burnout Inventory (MBI) and questions from previous studies on burnout, emotional exhaustion, depersonalization, and personal accomplishment. The survey was distributed from April 2020 to June 2020 to pharmacists across Australia through social media. The study compared the burnout rates among men and women in relation to gender role differences [8].

There were 647 responses that were analyzed and revealed that emotional exhaustion and depersonalization were higher than prior to COVID-19. When considering gender differences, male pharmacists reported higher depersonalization, more withdrawal, and increased cynicism as they felt more disconnected than prior to the pandemic (mean 9.2, SD=6.1, p=0.004) [8]. However, there were no differences in emotional exhaustion between men (mean 28.2, SD=14.6) and women (mean 28.8, SD=13.0, p=0.656), which emphasized the gender gap, with women more likely to have experienced isolation from social support networks, which increased stress and psychological burden. Likewise, differences in personal accomplishment were insignificant between men (mean 36.8, SD=7.7) and women (mean 36.6, SD=7.5, p=0.790). Regardless of gender differences, there were several factors that affected work, such as working overtime, limited medication supply, introduction to telehealth, unfamiliarity with personal protective equipment, increased administrative tasks due to limited staff, and patient incivility [8]. The researchers suggested that workplace changes may be addressed through interventions such as increasing the number of pharmacists on staff and increasing training and support for pharmacists.

As the personal lives of 87.2% of pharmacists were affected, the pandemic’s impact on psychosocial factors revealed that 83% of pharmacists felt isolated from family and friends, with 36.3% extremely concerned for their family’s health. Despite the negative factors indicated, 33.9% of pharmacists gained a better understanding of infection control, and 29.8% had an improved learning experience. The data yielded only 6.7% of pharmacists who believed the pandemic had no positive impact, with males more likely to report this (n=80, p=0.003) [8].

The study collected a convenience sample, so they did not report a traditional response rate, which served as a limitation. In addition, some pharmacists may have felt too burned out to complete the survey or contributed to selection and response bias due to reliance on self-reporting. During the study’s data collection, Australia’s borders were not fully shut down, which limited the study as it eliminated some populations and naturally skewed the participants’ mindsets on burnout. The researchers affirmed the importance of early identification of stressors that may contribute to burnout, as it can foster the implementation of self-care practices that would promote wellness in work and psychosocial elements of life [6].

Similarly, dos Santos et al. in Portugal aimed to determine the outcomes of burnout dimensions, assess the prevalence of burnout among pharmacists, and identify potential limits. The researchers analyzed variables of emotional exhaustion, depersonalization, and personal accomplishment in affecting burnout [6].

The methodology consisted of a cross-sectional survey conducted via Google Forms by the Portuguese Pharmaceutical Society (PPS) using the MBI and questions on work and social factors. The survey was divided into four segments: demographics, employment and workplace characterization, pandemic impact on labor activity, and burnout assessment. Though the survey was delivered to all 15,565 PPS members, the 1,362 respondents exceeded the minimal sampling size [6]. However, some limitations still existed as the study defined the pandemic period as three months, focused only on pharmacists in Portugal, potentially skewed results due to predominantly female respondents, and the disproportionate representation of pharmacists primarily practicing in community and hospital settings.

The dimensions of burnout outlined feelings of energy depletion and exhaustion, increased mental distance from one’s job or cynicism or negativity related to one’s job, and reduced professional efficacy. Reports from respondents revealed that burnout (53.2% of respondents reported experiencing burnout) was highest among pharmacists with shorter experience, who lacked confidence in providing care to patients with COVID-19, who lived alone, and who identified as male [6]. Likewise, emotional exhaustion and depersonalization were found to be the highest among pharmacists who lived alone, lacked confidence in caring for patients with COVID-19, worked on weekends, and increased work demands and the number of hours per week. The data indicated that pharmacists in direct patient care settings experienced burnout at a greater rate [6].

Also, personal accomplishment was reported to be significantly low among pharmacists during the pandemic for similar contributing reasons, such as time in business (χ2(1)=10.620, p=0.001), degree of confidence in providing pharmaceutical care to patients with COVID-19 (χ2(1)=31.562, p=0.000), completed training in COVID-19 (χ2(1)=7.507, p=0.006), and gender (χ2(1)=5.885, p=0.015). Reported low rates of personal accomplishment may have exacerbated the likelihood and risk of burnout among pharmacists working during the pandemic. Low personal achievement was reported among practicing pharmacists [6]. While burnout may not be a new challenge to the healthcare field, it has been accelerated by the effects of the pandemic. The researchers recognized the scarcity of research on burnout in pharmaceutical activity. The study's results indicated positive trends in burnout; however, this may not be applied to all occupational groups [6].

Hedima et al. investigated the risk of exhaustion, disengagement, burnout, and other associated factors to assess the impact of the COVID-19 pandemic on the mental health and well-being of pharmacists in Nigeria. The researchers contributed to data in resource-limited countries, like Nigeria, on the increasingly poor mental health and well-being of healthcare professionals. The study assessed three dimensions: exhaustion, negativism or cynicism, and decreased productivity under the focus of burnout [9].

The study's survey consisted of three sections. The first two sections addressed sociodemographic factors, mental health, and well-being. The third section explored the extent of burnout using a 16-item Oldenburg Burnout Inventory (OLBI) that measured disengagement and exhaustion. Data collection spanned from December 2020 to February 2021. A primary limitation of this study was its administration on WhatsApp, a social media platform not inclusive of all practicing pharmacists in Nigeria. In addition, this study only invited 17 actively practicing pharmacists from each of the 36 states in Nigeria who belonged to at least one of the pharmacists' WhatsApp groups, totaling 612 pharmacists invited to participate. The Research Review Board of the Faculty of Pharmaceutical Sciences at Gombe State University in Nigeria approved this methodology. Despite these limitations, the study had a high response rate of 69.6% of pharmacist respondents (426 pharmacists completed the survey) out of 612 invited pharmacists [9].

A comparison of pre- and post-pandemic reporting revealed the pandemic’s substantial effects on the mental health and well-being of pharmacists. The researchers recognized that the rate of burnout had increased over the past five years in Nigeria. The pandemic had a significantly high impact on 27.7% of respondents and a partial impact on 43.4% of respondents. A majority of the pharmacist respondents stated that they had growing worries about the quality of patient care and services they could provide at the workplace. The study’s results revealed that 54.9% of respondents did not take time off during the pandemic. Of these respondents, 80% of those who were unable to take time off had the highest risk of burnout. A large portion of pharmacists, especially in the hospital setting of direct care, stated they had considered leaving the profession (43.8%). A majority of respondents (75.6%) met the high risk of exhaustion and disengagement, which is attributed to burnout [9]. The OLBI scores depicted a high risk of exhaustion and disengagement or burnout among 66.2% of the 75.6% of pharmacist respondents who met the criteria. Despite the stressors, 78.2% of respondents reported enjoying their work, which potentially illustrated high personal accomplishment and efficacy. Burnout was reported to be higher among those who do not enjoy work (96%) than compared to those who do enjoy work (62.4%) [9]. The researchers analyzed the realities of young and inexperienced pharmacists who felt feelings of uncertainty and incompetence when adapting to rapid workplace changes during the pandemic. Of the respondents under the age of 36 years old, 59% identified with factors associated with burnout, whereas only 31.9% of respondents (ages 36-55 years old) and 9% of respondents above 55 years old identified with burnout factors [9]. These burnout factors included a decline in productivity, job dissatisfaction, and a tendency to change positions or leave a current job [9]. Furthermore, the study suggested that job satisfaction increases with age and experience, which may reduce the risk of burnout by decreasing exhaustion and disengagement. The researchers noted the importance of strategies from employees, government, and organizations that can help improve conditions at the workplace. These strategies include participation in managing workplace stress early in one's career, workplace mentoring programs for pharmacy students and young pharmacists, and preparation for real-world practice to improve the overall well-being of pharmacists.

National: USA

Meanwhile, in the USA, Golbach et al. sought to explore the prevalence of burnout among hematology-oncology pharmacists and the factors associated with an increase in burnout. In February 2021, the American Society of Clinical Oncology (ASCO) published a five-year plan addressing provider burnout and well-being by focusing on engaging clinical well-being, broadening resources, promoting research to identify needs, and making recommendations to optimize care delivery while considering threats to patient and provider wellness [2]. This narrative literature review provides a starting point and foundation for understanding ASCO's call to action within the context of practice. This review examines how initiatives can be applied to improve the well-being of pharmacists, particularly in states like Kansas, where the urban-rural dichotomy presents unique challenges. By contextualizing ASCO's plan within rural settings, the manuscript demonstrates the value of targeted interventions and highlights the importance of addressing both urban and rural healthcare environments to enhance overall provider well-being and care delivery.

A cross-sectional online survey was distributed to members of the Hematology/Oncology Pharmacy Association (HOPA) between October and November 2020. The methodology focused on the assessment of well-being, burnout, and sociodemographic and occupational factors among hematology-oncology pharmacists through questions derived from the MBI and Well-Being Index [2]. Limitations of the study included a low participation rate of 20.3%, a single specialty of pharmacists, a survey length of 58 questions (limitations identified by researchers themselves), and differences in demographics between the respondents and the general membership of HOPA.

Several characteristics of burnout were emphasized, including energy depletion, exhaustion, cynicism, negativity, and reduced professional efficacy. Results from the MBI reported that 61.8% of respondents experienced high burnout (95% CI (57.7-65.9)). Likewise, 57.9% of respondents reported feeling emotional exhaustion as many pharmacists were working on average 48.6 hours per week, which included 7.5 more hours of administrative work (OR=2.40, 95% CI (1.52-3.78), p<0.001). The multivariable analysis revealed factors associated with increased risk of burnout, including the following sociodemographic and occupational factors: increasing age, increasing workload, working overtime, more administrative work, being unaware of wellness support programs, and overall decreasing wellness due to the pandemic. The study relayed how pharmacists with burnout were four times as likely to have made medication errors (27.6% vs. 8.1%, p<0.001) and/or intended to leave their current position (26.8% vs. 8.1%, p<0.001) during the pandemic [2]. The researchers identified several key risk factors for burnout among healthcare providers, including a high workload, inadequate staffing, a lack of support, and insufficient resources. By targeting these risk factors, prevention strategies can be developed and implemented to mitigate burnout and ensure the sustainability of the current workforce.

Jones et al. studied the prevalence of burnout and secondary traumatic stress (STS) in health-system pharmacists (HSPs) during the COVID-19 pandemic. The methodology consisted of a cross-sectional online survey administered to HSPs across the USA. The 62-item questionnaire covered the following topics: demographics, employment characteristics, COVID-19-related questions, a survey for self-reporting burnout, and the Professional Quality of Life (ProQOL). The survey was administered relatively early in the pandemic, which served as a limitation in an assessment of the true effects of burnout [4].

Despite its lengthy survey of 62 questions, the survey had a 77% response rate. Their results revealed that 47% of the 484 HSPs identified with current burnout and 81% with a history of burnout. The ProQOL scoring indicated a higher prevalence of moderate to high burnout (65.3%) than compared to the respondents’ self-reports. Similarly, 51.4% of respondents identified with a moderate to high probability of STS. Compassion satisfaction was moderate to high among 99.4% of the respondents. The researchers identified that the increase in burnout and STS because of the pandemic negatively impacted workplaces due to the increase in compassion fatigue. A total of 46.7% of respondents experienced an increase in their work hours, and 34% lost childcare services, which affected their ability to work. The burnout characteristics attributed to changes in personal life and workplaces due to the pandemic increased the likelihood of stress disorders, as evidenced by the increase in STS (51.4%) and emotional exhaustion (70%).

Emotional exhaustion contributes to compassion fatigue. Burnout and STS displayed an inverse relationship with compassion satisfaction, as the likelihood of compassion satisfaction was moderate to high (99.4%) [4]. The study emphasized how the development of burnout and STS may lead to consequences in the workplace; thus, further research on the impact of burnout and STS is needed.

Mohammad et al. described the changing patterns of burnout and STS prevalence among pharmacists throughout the pandemic, from the early stages to 20 months later. The researchers sought to acquire more evidence on the prevalence of burnout and STS as the increasing demands of the pandemic brought about more stress, frustration, exhaustion, and cynicism among healthcare workers [10].

A cross-sectional online survey was administered to five communities that include pharmacists practicing in the USA health system through the American Society of Health-System Pharmacists (ASHP) between April and May 2020 for early group data collection and again between October 2020 and December 2021 for 20-month group data collection. While assessing the study’s methodology, it was noted that the questionnaire was extensive with 62 questions and included many external factors such as salary, loans, sleep schedules, etc.; however, the large participation group of 1,126 HSPs through the ASHP was a key strength of the study. The survey asked questions relating to COVID-19, self-reporting of the severity and prevalence of the individual’s burnout, and the ProQOL, which helped assess compassion fatigue and satisfaction [10].

Of the 1,126 HSPs, 69% of the participants reported feeling more burned out 20 months into the pandemic compared to the 47.7% of participants in the early group. Based on the ProQOL, 20 months into the pandemic, 83% of pharmacists identified burnout, 63% with STS, and 99% with compassion satisfaction. The results revealed a significant increase (p<0.001 for all three variables) in burnout and STS from the early group to the 20-month group. A constant theme was evidenced by pharmacist respondents from both groups identifying with a moderate-high probability of compassion satisfaction (99%) [10]. Compassion satisfaction was a positive factor in increasing the chance of overcoming compassion fatigue related to the pharmacists’ job demands. However, when taking a closer look, it was discovered that there were more respondents in the 20-month group who identified with a low likelihood of compassion satisfaction. Compassion fatigue is characterized by burnout and STS; thus, the prolonged pandemic stress may have affected satisfaction as it contributed to an increase in burnout and STS likelihood among the 20-month group [10].

The study suggested that the development of burnout and STS led to work-related consequences among pharmacists, such as a decrease in productivity, an increase in medical errors, disruption of quality patient care and relationships with colleagues, and physical and mental health. The increase in burnout has worsened the mental health of pharmacists, specifically those in direct patient care, leading to a rise in depression, anxiety, and post-traumatic stress syndrome [10]. This called for further studies on the key factors that led to the increase in burnout prevalence as the pandemic progressed rather than at the beginning. It highlighted the need for further research on effective interventions to address the well-being of pharmacists.

Interestingly, Ruble et al. sought to determine the correlation that exists between pharmacists’ emotional intelligence and work-related components such as occupational stress, job performance, and psychological affective well-being. A Qualtrics survey was administered through e-mail to active Florida-licensed pharmacists from the Florida Board of Pharmacy. The 40-question survey covered the following topics: four facets of emotional intelligence, demographics, occupational stress, job performance, and psychological affective well-being. The survey design incorporated various point scales (e.g., a seven-point Likert scale ranging from strongly disagree (1) to strongly agree (7)) for each topic of assessment. The data was collected between October 2020 and December 2020, during the height of the COVID-19 pandemic in Florida. A primary limitation of the study was the low response rate of Florida pharmacists’ participation, 3% (942/31461) [11].

The study revealed that higher emotional intelligence is correlated with less stress (r=2.75, r=2.9), higher job performance (r=4.1, r=4.18), and higher psychological affective well-being (r=3.54, r=3.52) for men and women, respectively. Though female pharmacists reported higher stress, gender did not significantly affect job performance or psychological affective well-being. The primary employment setting influenced the stress, job performance, and psychological affective well-being of pharmacists. While community-based pharmacists reported higher stress, hospital pharmacists reported higher job performance (r=4.28) and psychological affective well-being (r=3.6) than community-based pharmacists, respectively (r=4.0, r=3.3) [11]. The researchers suggested that higher emotional intelligence supported the overall wellness of pharmacists during the pandemic. The study's findings highlight the crucial role of emotional intelligence in mitigating stress and enhancing job performance and psychological well-being among pharmacists. Understanding these correlations is vital for practical applications in the workplace. Emotional intelligence can be leveraged to develop targeted training and support programs that address stress management, improve job performance, and boost overall well-being. For instance, training programs that focus on developing emotional intelligence skills could help pharmacists better manage the pressures of their roles, particularly in high-stress environments like community-based settings. Additionally, the variation in stress, job performance, and well-being between community-based and hospital pharmacists underscores the need for tailored interventions that address the unique challenges of each employment setting. By prioritizing emotional intelligence in professional development, healthcare organizations can enhance the resilience and effectiveness of their pharmacy staff, ultimately leading to better patient care and reduced burnout. This approach is not only important for individual wellness but also for maintaining a competent and effective pharmacy workforce during challenging times.

Regional: Midwestern USA

Focusing on the Midwestern USA, McQuade et al. analyzed burnout prevalence among HSPs and ambulatory care pharmacists (ACPs) during the COVID-19 pandemic. The researchers defined burnout as high emotional exhaustion and depersonalization with low personal accomplishments from work. The study was carried out to contribute to the lack of research on burnout prevalence among HSPs and ACPs during the pandemic [5].

The methodology employed a survey administered at the University of Illinois Hospital and Health Sciences System and Rush University Medical Center. The questionnaire consisted of the OLBI and MBI, along with questions on demographics, burnout contributing factors, mitigation strategies, and changes in burnout due to the pandemic. The researchers evaluated high burnout through data on exhaustion scores, disengagement, emotional exhaustion, and depersonalization [5].

The survey had a participation rate of 60% HSPs (148/246). The OLBI and MBI results revealed that the prevalence of burnout was high among both HSPs (87.1%) and ACPs (88.4%) at similar rates (p=0.85). The most common causes of burnout identified in this study were scheduling concerns, staffing issues, precepting requirements, and patient needs for quality care.

McQuade et al. estimated nine out of 10 HSPs were at high risk for burnout, which correlated with increased medical errors, patient harm, frayed interpersonal relationships, reduced patient satisfaction, and reduced healthcare access at work [5]. Pharmacists’ most reported coping strategies were spending time with family and friends, sleeping, exercising, and engaging in recreational and relaxation activities. Though pharmacists employed coping strategies that were helpful in stress reduction and improving health, they served as poor buffers for work-related burnout. For pharmacists who met the burnout criteria, the coping strategies served a limited role in reducing burnout as they did not address the underlying causes and contributors of burnout. A primary limitation of the study was that the sampling pool consisted only of HSPs practicing direct patient care at two academic health systems in Chicago, Illinois (the University of Illinois Hospital and Health Sciences System and Rush University Medical Center), which diminishes its ability to be extrapolated to the national or international levels. The researchers determined that efforts to reduce burnout must come from an organizational level, as personal coping efforts were not sufficient.

Kansas

Furthermore, the authors of the present manuscript conducted a joint research study between the University of Kansas School of Pharmacy and the Kansas College of Osteopathic Medicine, which addressed a gap in the literature [12]. In the survey study, 107 pharmacists participated, which aimed to explore the psychological well-being of Kansas pharmacists during the COVID-19 pandemic, offering recommendations for employers and policymakers based on statistical analysis. Pharmacists in Kansas found themselves overwhelmed by a variety of challenges during the pandemic, including an increased influx of COVID-19-related inquiries (5.84%, n=18), heightened instances of patient harassment (10.39%, n=32), and disruptions caused by political activities (7.11%, n=27) [12]. To address these issues and based on respondent feedback, it is recommended that pharmacists be provided with dedicated counselors and offices to guide patients, particularly those dealing with heightened disease states or mental health effects linked to their pharmacy care [12].

Discussion

Comparative Analysis of International, National, and Regional Studies

Across the various studies included in this review (international, national, and regional), it has been recognized that pharmacists faced similar issues as other healthcare professionals during the COVID-19 pandemic [5-7,9]. This section of the literature review presents a comparative analysis of all the studies included in this review, despite geographical differences. Studies suggest the pressure on the pharmacy workforce has been exacerbated following the pandemic [4,6-9], even though pharmacists had already been experiencing burnout before the pandemic [9]. The findings from all the studies mentioned in this review revealed common themes of burnout and work and psychosocial factors affecting the well-being of pharmacists.

Burnout: Burnout was defined as feelings of energy depletion [2,8], a high risk of exhaustion and disengagement [8,9], and reduced professional efficacy [2,8]. Internationally, burnout is considered a work-related issue due to unmitigated long-term workplace stress from overload and overwork [6,8,9]. In the national and regional studies conducted in the USA that were included in this review, there were no statistically significant gender differences in burnout and well-being [8,11,12]. However, a study conducted in Nigeria, which assessed burnout among practicing Nigerian pharmacists, concluded that there was a significantly higher risk of exhaustion related to burnout in female respondents compared to male respondents [9]. An international study in Australia identified that gender-specific differences have been inconsistently reported and require further investigation [8]. The impact of the pandemic on the healthcare system was a higher incidence of burnout and an increase in mental health conditions, requiring further advocacy for pharmacists' mental health and well-being [9].

Work and psychosocial factors: The COVID-19 pandemic increased workload and stress for practicing pharmacists at the international [6,8,9], national [2,4,10,11], and regional [5,12] levels. These studies shared similar workplace factors that impacted well-being, such as increased workload, increased hours worked per week, inadequate staffing, scheduling issues, a demanding work environment, learning to work via telehealth, and a lack of resources [2-12]. Increased burnout has been linked to the lack of social support due to the pandemic [3]. Apart from occupational stressors, several other social stressors affected pharmacists’ well-being, which included the following: family, childcare, personal health, and financial and housing instability [2,5,6-9,12].

Overall, there was a lack of a healthy work-life balance for practicing pharmacists across the world [4,6,9,10,12], which led to an increased proportion of individuals experiencing mental health challenges during the COVID-19 pandemic [9,12].

Recommendations for Improving the Well-Being of Pharmacists

During the pandemic, pharmacists suffered from reduced job satisfaction, decreased productivity, a lack of interprofessional teamwork, and a negative impact on physical and mental health [3]. The American College of Clinical Pharmacists recognized the urgency of a call to action for addressing burnout in the USA’s healthcare system through an applicable and adaptive approach for healthcare professionals. To cater such efforts toward pharmacists, a collective understanding of shared challenges, experiences, and perspectives on the workplace and mental health must be achieved among various healthcare professionals. Interprofessional collaboration is necessary to provide sustainable, effective organizations and individual interventions [3].

Counseling: It is recommended that pharmacists be provided with dedicated counselors and offices to guide patients, particularly those dealing with heightened disease states or mental health effects linked to their pharmacy care. Professionals in psychological, therapeutic, and counseling roles can raise awareness about their specialties within the local community by visiting pharmacies to share information on the types of support and care they offer, such as psycho-oncology, health psychology, and palliative and hospice psychology [12]. This may entail offering telemental health, non-standard therapy hours and locations, such as early morning or late evening, and traveling to those seeking support in rural settings [12]. Psychologists, therapists, and counselors serve an integral role in interprofessional collaboration as advocates for empowering individuals to take agency for their personal well-being.

Workplace: Additionally, establishing a supportive work environment that prioritizes mental health is crucial, as psychological well-being is equally important. Identifying well-being as an organizational priority can encourage healthcare professionals to prioritize their own well-being through self-care practices that engage in mindfulness and stress reduction techniques and invite support from friends, family, and/or professional counselors. Open communication fosters an environment where healthcare professionals can express their feelings and concerns without fear of judgment, facilitating sharing experiences and seeking support. Regular debriefing sessions and peer support networks create safe spaces for discussing challenging cases and reducing feelings of isolation. Active engagement of various healthcare professionals can diminish burnout by promoting collaboration, interpersonal development, and a team culture [3].

Interprofessional collaboration: Effective interprofessional collaboration is essential to implementing interventions for both individuals and reinforcing well-being policies aligned with support and care [3]. Organizational interventions, including mental health resources and training, prove more effective than individual-focused efforts [13]. Workshops on stress management, resilience, and self-care equip healthcare professionals with practical skills to cope with work-related stressors [14]. While the impact of resilience training on team members is inconclusive, offering optional choices, like mindfulness training, is a reasonable approach to preventing resentment and disengagement. Individual interventions can be integrated into organizational initiatives, such as Schwartz Center Rounds, providing clinicians with opportunities to discuss social and emotional challenges [15]. Regular mental health screening and assessments help identify early signs of burnout, enabling timely support. Nationally, employee assistance programs offer confidential counseling services, connecting professionals to licensed therapists nationwide, which can help ameliorate the growing prevalence of burnout in the USA. A Resource Compendium for Health Worker Well-Being by the National Academy of Medicine provides evidence-based measurement tools. Professionals are urged to prioritize their well-being through self-care practices, including relaxation, exercise, healthy eating, sufficient sleep, and engaging in hobbies and social connections [16].

Each professional member of a patient's healthcare team plays an indispensable role in the delivery of quality patient care. While professionals face nuanced challenges in their respective fields, recognizing the interconnectedness of their colleagues allows for a more comprehensive approach to addressing the multifaceted issues affecting the well-being of one another [17]. By acknowledging the unique perspectives and challenges inherent in each role within the healthcare team, professionals can collaboratively implement comprehensive strategies that enrich the overall mental health and well-being of the entire healthcare community. This includes the unique perspectives and challenges of practicing pharmacists. A sense of unity not only enhances the ability to navigate current challenges but also reinforces the shared commitment to providing exceptional care to patients [18].

Practical Implications

This review aimed to share the current research on the perceptions and experiences of practicing pharmacists during the COVID-19 pandemic and emphasize the necessity for further research in this area of study. As evidenced by the studies mentioned in this review, there is a need for sustainable mental health interventions to improve the well-being of practicing pharmacists. These recommendations may be provided to employers and policymakers in efforts to engage in effective change to further support the mental health of pharmacists [3,12].

Limitations

Several limitations existed in this review. Many studies shared a broad definition of burnout, which entailed increased exhaustion and disengagement along with reduced professional efficacy and unmitigated stress [2-11]. However, these studies may not have considered other factors that impacted burnout apart from workplace changes and family commitments. Also, many of these studies were conducted in resource-rich areas, except for Nigeria [9] and rural parts of Kansas [12], which may have played a role in the workplace struggles practicing pharmacists faced in terms of resource availability and patient demands. In terms of accessibility to the participants in these studies, many studies used electronic platforms that may not have allowed for a complete representation of the practicing pharmacists in the area (WhatsApp in Nigeria [9], Google Forms [6], Qualtrics in Kansas [4,12]).

Future Research

Though the current literature provides insight into the impact of the COVID-19 pandemic on practicing pharmacists' well-being, there are several opportunities for prospective studies to further investigate this impact. An in-depth analysis of pharmacists' perspectives can serve to thoroughly understand one's mental health challenges and overall well-being during the pandemic's drastic changes and struggles. It is critical to investigate the long-term effects of the pandemic on pharmacists' well-being worldwide. Future research should aim to address gaps in research on risk factors for burnout, interventions for practicing pharmacists, and strategies to reduce the long-term impacts of the pandemic on pharmacists' work-life balance and well-being.

## Conclusions

Amidst the prevailing challenges facing healthcare professionals, it is imperative to underscore the solidarity inherent in the healthcare community. Collaborative efforts among healthcare professionals contribute to a resilient and cohesive healthcare system. These efforts require a strategic commitment from the healthcare system, as the well-being of pharmacists cannot be adequately addressed by a pharmacy workplace, organization, or institution alone. Despite current research efforts, further research is needed to explore the mental health challenges of practicing pharmacists worldwide. Prospective studies must seek to promote collaboration, stress management, and overall well-being to ensure sustained work-life integration for pharmacists. The call to action is clear, with future research and interventions promoting collaboration, stress management, and work-life balance for pharmacists.
